# Extremes in Healing

**Published:** 1888-08

**Authors:** 


### HALL'S JOURNAL OF HEALTH

TRUTH DEMANDS NO SACRIFICE; ERROR CAN MAKE NONE.

VOL. 35. AUGUST, 1888. NO. 8.

### EXTREMES IN HEALING.

There has ever been a tendency among new converts to go to extremes, especially in respect to religion, or matters into which religion enters as one of the elements. Oftentimes their over-zeal assumes a fanatical turn, from which, a recovery is seldom had. This is as plainly exemplified in respect to methods of healing as in matters of minor importance. The oldest of our established schools of medicine, that which claims as its heritage the transmitted knowledge of the earliest empirics, down to the latest allopathist, confined its teachings to the physical side of being, rating human disorders no otherwise than those which affect the lower animals in the effort to heal a wound or eradicate disease.

Now, to the appreciative mind it would seem quite as inconsistent to treat a patient as one would treat a dumb animal, as it would to attempt to delude him into the belief that his fractured limb was a mental hallucination. His faith may be as a mountain, yet the limp member must be carefully set, splinted and bandaged before the process of healing can go on.

We have, heretofore, asserted in these columns that no physician who treats his patients from a purely materialistic standpoint is capable of intelligibly diagnosing a class of diseases largely induced by mental distractions, or ministering to their relief by the wisest methods, and the reverse proposition is equally true of the exclusively mind curists, who regard the physical as unworthy of consideration. It is indeed this ignoring by the old school of the very decided part which the mind plays in all our ailments in inducing and keeping alive the disorders that prey upon the body, that has opened wide the door to the extremes of treatment now flashed into sudden and unsubstantial favor under the names of the mind, the prayer and the faith cure; fruit of the same ephemeral stock, which has only to be tested to discover its imperfections. Let us bring face to face these two extremes which, at present, rival each other with that unreasoning opposition which admits of no compromise, no blending into a harmonious whole, neither of which can ever reach anything like perfection for want of the other as a helpmate. On the one hand the mind is troubled by some overpowering sorrow or misconception, and needs to be sustained and reinvigorated, and you physic and fret the body with drugs and nostrems ; on the other, the body is diseased, crushed and broken down, and you address yourself solely to the mind, declaring all sickness a delusion ! There is greater wisdom in the following lines than in many a labored treatise of the schools :

*Macbeth.*—"Canst thou not minister to a mind diseased,
Pluck from the memory a rooted sorrow,
Raze out the written troubles of the brain,
And with some sweet oblivious antidote
Cleanse the stuff'd bosom of that perilous stuff,
Which weighs upon the heart ?

*Doctor.*—Therein the patient must minister to himself.
*Macbeth.*—Throw physic to the dogs, I'll none of it."

The influence of the mind over the vital functions has been frequently illustrated. A criminal condemned to undergo the death penalty after being told that his executioners had contrived a comparatively painless death for him, was bound upon a stretcher, a pretense of puncturing an artery made, and water allowed to drip, drop by drop, into a receptacle, in his hearing. Under the impression that he was being slowly bled to death, the convict actually expired under the operation. So much for the mind ; now, supposing the subject had merely been bled to exhaustion, is it reasonable to believe that any effort upon the mind could have restored his lost energies ? Men have been known to imagine themselves in the most absurd conditions, even to being a tool or a household utensil, an hallucination of which, it was found quite impossible to disabuse them.

In all similar instances it would be as absurd to rely wholly upon physical remedies to effect a cure as it would to appeal solely to a mind. Both the mental and physical should be considered in the treatment, both should be operated upon, and herein lies the vantage ground between the two extremes, neither of which should, in any event, be unmindful of the benefits to be derived from a recognition of the other. No amount of physic will cure "a mind diseased, and no persistency in prayer will set a broken leg.

